# Clinical and immunological comparison of COVID-19 disease between critical and non-critical courses: a systematic review and meta-analysis

**DOI:** 10.3389/fimmu.2024.1341168

**Published:** 2024-04-16

**Authors:** Mojtaba Hedayati-Ch, Hadi Sedigh Ebrahim-Saraie, Arash Bakhshi

**Affiliations:** ^1^ Department of Microbiology, Virology and Microbial Toxins, School of Medicine, Guilan University of Medical Sciences, Rasht, Iran; ^2^ Microbial Toxins Physiology Group (MTPG), Universal Scientific Education and Research Network (USERN), Rasht, Iran; ^3^ Member of Research Committee, School of Medicine, Guilan University of Medical Sciences, Rasht, Iran

**Keywords:** COVID-19, critical, co-infection, mortality, sever

## Abstract

**Introduction:**

Acute Respiratory Syndrome Coronavirus 2 (SARS-CoV-2), which appeared in 2019, has been classified as critical and non-critical according to clinical signs and symptoms. Critical patients require mechanical ventilation and intensive care unit (ICU) admission, whereas non-critical patients require neither mechanical ventilation nor ICU admission. Several factors have been recently identified as effective factors, including blood cell count, enzymes, blood markers, and underlying diseases. By comparing blood markers, comorbidities, co-infections, and their relationship with mortality, we sought to determine differences between critical and non-critical groups.

**Method:**

We used Scopus, PubMed, and Web of Science databases for our systematic search. Inclusion criteria include any report describing the clinical course of COVID-19 patients and showing the association of the COVID-19 clinical courses with blood cells, blood markers, and bacterial co-infection changes. Twenty-one publications were eligible for full-text examination between 2019 to 2021.

**Result:**

The standard difference in WBC, lymphocyte, and platelet between the two clinical groups was 0.538, -0.670, and -0.421, respectively. Also, the standard difference between the two clinical groups of CRP, ALT, and AST was 0.482, 0.402, and 0.463, respectively. The odds ratios for hypertension and diabetes were significantly different between the two groups. The prevalence of co-infection also in the critical group is higher.

**Conclusion:**

In conclusion, our data suggest that critical patients suffer from a suppressed immune system, and the inflammation level, the risk of organ damage, and co-infections are significantly high in the critical group and suggests the use of bacteriostatic instead of bactericides to treat co-infections.

## Graphical Abstract

Summary of the comparison of two clinical course of COVID-19

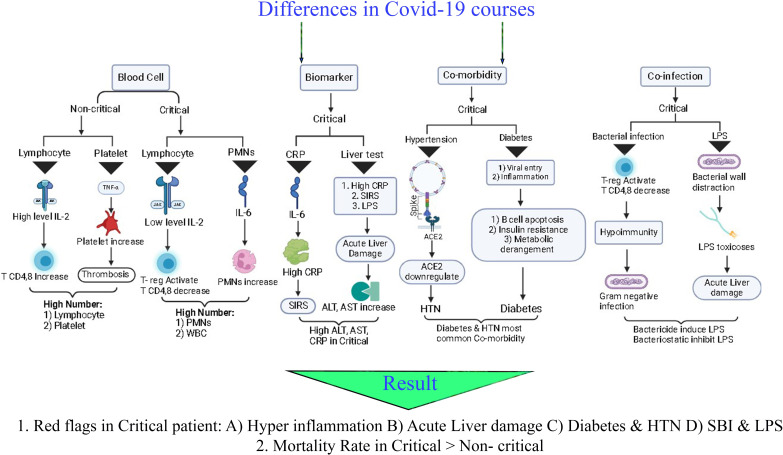

## Introduction

The SARS Coronavirus 2 (SARS-CoV-2) originated in China and spread to most countries worldwide in 2019. Generally, more than 200 million confirmed cases and more than 4 million deaths have been reported. SARS-CoV-2 is more infectious than SARS-CoV (1–3). The new Coronavirus is classified into critical and non-critical cases based on symptoms. Description of critical patients require mechanical ventilation and intensive care unit (ICU) admission, and Non-critical patients don’t require mechanical ventilation and ICU admission (3, 4). Accordingly, comparing critical and non-critical groups can describe the difference between the presence and absence of co-infection. After three years of the first appearance of COVID-19, researchers examined essential factors for evaluating COVID-19 disease.

As a first step, the blood cell count was evaluated. Some papers suggest that the number of white blood cells (WBCs), lymphocytes, and platelets may vary as a result of COVID-19, including critical, mild, moderate, and severe cases (5, 6). The difference between these cells may show the infection and inflammation in critical and non-critical groups (7, 8). Another factor associated with COVID-19 may be enzymes and proteins. Alanine aminotransferase (ALT), Aspartate aminotransferase (AST), and C reactive protein (CRP) levels were inconsistent between critical and non-critical groups. The level of these markers can determine the prognosis of the two groups (9, 10).

Underlying diseases such as high blood pressure, diabetes, cardiovascular disease, and dyslipidemia can play a crucial role in the clinical course of COVID-19 patients. Critical and non-critical patients exhibit varying levels of comorbidities; these have a different impact on morbidity (11). Moreover, there is evidence to suggest that the mortality rate can be affected by comorbidities (12, 13).

Co-infection is an essential factor in morbidity and mortality. Globally, the prevalence of bacterial co-infection in COVID-19 patients is unknown and different micro-organisms lead to co-infection (14). Most of the organisms differ in their distribution in different organs, such as the respiratory, blood, and urinary tracts (15). As a result, the reaction of critical and non-critical groups to co-infections will be considerably variable. Bacterial co-infection plays a vital role in the clinical course of the COVID-19 disease, which can be treated with various antibiotics.

The mortality rate of COVID-19 disease can be affected by factors such as blood cell count, blood markers, comorbidities, and co-infections (4). Therefore, the mortality rate can differ between critical and non-critical groups. In this study, blood markers, comorbidities, co-infections, and their relationship to mortality rates were compared between critical and non-critical patients.

## Method

### Search strategy

We reported this systematic review meta-analysis using the Preferred Reporting Items for Systematic Reviews and Meta-Analyses (PRISMA) guidelines. The studies were identified using the following PICOS principle: **P**atients = Patients with COVID-19, **I**ntervention = dividing patients into critical and non-critical based on ICU admission and mechanical ventilation, **C**ontrol, **O**utcome = Comparison of immunological and clinical factors between critical and non-critical groups, **S**tudy design = case-control, prospective or retrospective studies (16). We used Scopus, PubMed, and Web of Science databases for our systematic search. The search terms used in the database were included (“COVID-19” OR “SARS COV-2” OR “Coronavirus infection”) AND (“critical” OR “Non-sever”) AND (“non-critical”) AND (“Co-infection” OR “Secondary infection” OR “bacterial infection”). We searched English publications and stored and checked articles using Endnote software as a citation manager. All selected articles were published in the 2019 to Jan 2022 date range. We reviewed the search results’ titles, abstracts, and full text for screening and study selection based on the inclusion criteria. Inclusion criteria include any original study that evaluated differences in 1) blood cells and blood markers, 2) bacterial co-infection, 3) Comorbidities, and 4) mortality Rate between critical and non-critical COVID-19 patients. Viral co-infection, case reports, reviews, and duplicate studies are generally excluded from this systematic review.

### Quality assessment and data extraction

Rayyan platform was used for screening and data extraction of included studies. Using the nine-point Joanna Briggs Institute critical appraisal checklist for studies, two researchers conducted the quality assessment (A. B and M. H) and disagreements were resolved by consensus (H. S). The included studies met more than half of the quality assessment parameters. Based on **Table 1**, we (A. B. and M. H.) extracted the publication year, country, the number of patients, the clinical course (ICU admission or mechanical ventilation), and physiological data from the studies. The prevalence of bacteria and co-infections were determined using nasal and pharyngeal swabs, blood serum, and urine analysis samples for the respiratory, bloodstream, and urinary systems, respectively in selected studies. As a part of our investigation, the following information was obtained: publication year, study design and research question, number of articles, number of each type of study, language, and country of study, device used, patient characteristics, and statistical methodology.

**Table 1:** The Main Characteristics of Studies Included in the Meta-analysis.

Study name	Year	Country	Study design	Critical (%)	Non-Critical (%)	JBI score (RoB)	References
Tao Zuo	2020	China	Prospective	2 (20%)	8 (80%)	5 (Moderate)	(17)
Tang	2019	China	Cohort	18 (48%)	19 (52%)	4 (Moderate)	(10)
Shi	2020	China	Case-control	6 (30%)	14 (70%)	8 (Low)	(7)
M. McKeigue	2021	Scotland	Case-control	702 (16.5%)	3533 (83.4%)	6 (Moderate)	(18)
Liaqat	2021	Pakistan	Retrospective	57 (28.3%)	144 (71.6%)	4 (Moderate)	(4)
Liu	2021	China	Retrospective	16 (18.5%)	69 (81.1%)	9 (Low)	(9)
Tian	2020	China	Retrospective	45 (50%)	45 (50%)	8 (Low)	(12)
Jieyu He	2020	China	Prospective	49 (43.7%)	63 (56.2%)	7 (Low)	(19)
Yuan Cen	2020	China	Retrospective	22 (10%)	200 (90%)	6 (Moderate)	(20)
Jianfeng Wu	2020	China	Retrospective	697 (30%)	1690 (70)	9 (Low)	(21)
Fukushima	2021	Japan	Retrospective	41 (18%)	193 (82%)	8 (Low)	(22)
Wang	2020	China	Retrospective	50 (41%)	73 (59%)	8 (Low)	(13)
Liu	2020	China	Retrospective	30 (32%)	65 (68%)	7 (Low)	(23)
Cheng	2020	China	Retrospective	52 (21%)	200 (79%)	6 (Moderate)	(24)
Zhihua Lv	2020	China	Retrospective	84 (42%)	115 (58%)	8 (Low)	(25)
Caméléna	2021	France	Prospective	43 (100%)	–	5 (Moderate)	(26)
Contou	2020	France	Retrospective	92 (100%)	–	6 (Moderate)	(27)
Rothe	2020	Germany	Retrospective	–	56 (100%)	5 (Moderate)	(28)
THOMSEN	2021	Scandinavian	Cohort	34 (100%)	–	3 (High)	(29)
Amaravati	2021	Indonesia	Retrospective	52 (56%)	40 (44%)	3 (high)	(30)
Yang	2021	China	Retrospective	58 (60%)	38 (40%)	8 (Low)	(31)

RoB, Risk of Bias.

### Data analysis

The statistical analysis and construction of graphs were performed with a comprehensive meta-analysis (CMA) version 3 (Biostat Inc., Englewood, NJ) with a random effect model plotted on forest plots since this model is more reasonable in the presence of heterogeneity than the fixed model. The pooled standard difference in mean with 95% CI gave the summary estimate. To test heterogeneity, we used the I-squared (*I^2^*). Visual bias was assessed using a funnel plot, and Egger’s regression test confirmed it (p < 0.05 was considered a statistically significant publication bias).

## Result

### Search outcome and study characteristics

**Figure 1:**
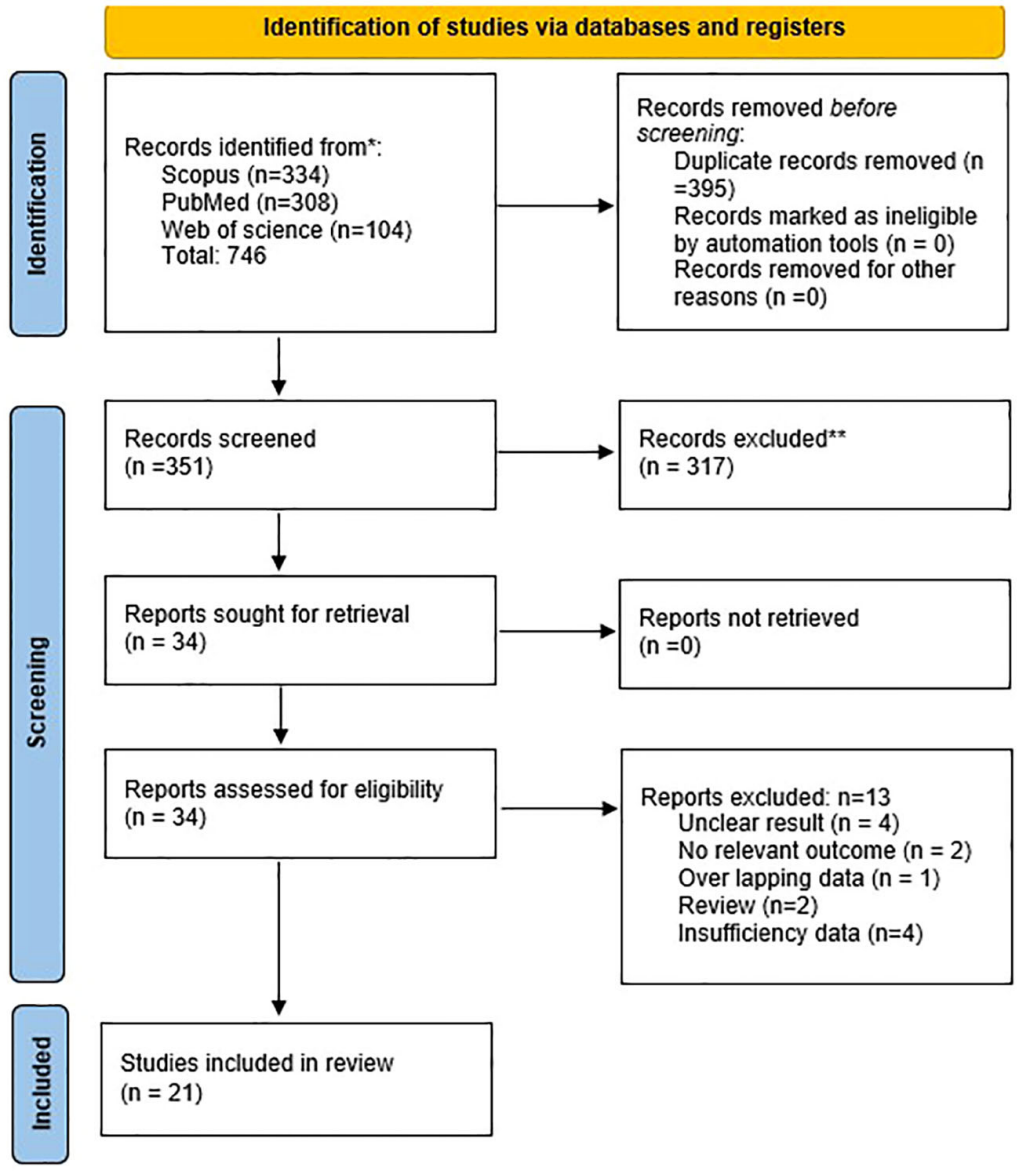
PRISMA flow chart of study selection.

Considering the objectives of this study, we identified 746 publications in Scopus, PubMed, and Web of Science databases. After removing duplicate studies and screening based on inclusion and exclusion, 21 publications were eligible for full-text examination (**Figure 1**). Studies have been conducted in the following countries: China (13), France (2), and one study from each of the following: Germany, Scotland, Pakistan, Japan, Scandinavian, and Indonesia. Among the studies, there were 14 retrospective studies, three prospective studies, two case-control studies, and two cohort studies.

### Comparison of blood cell count between critical and non-critical groups

**Figure 2:**
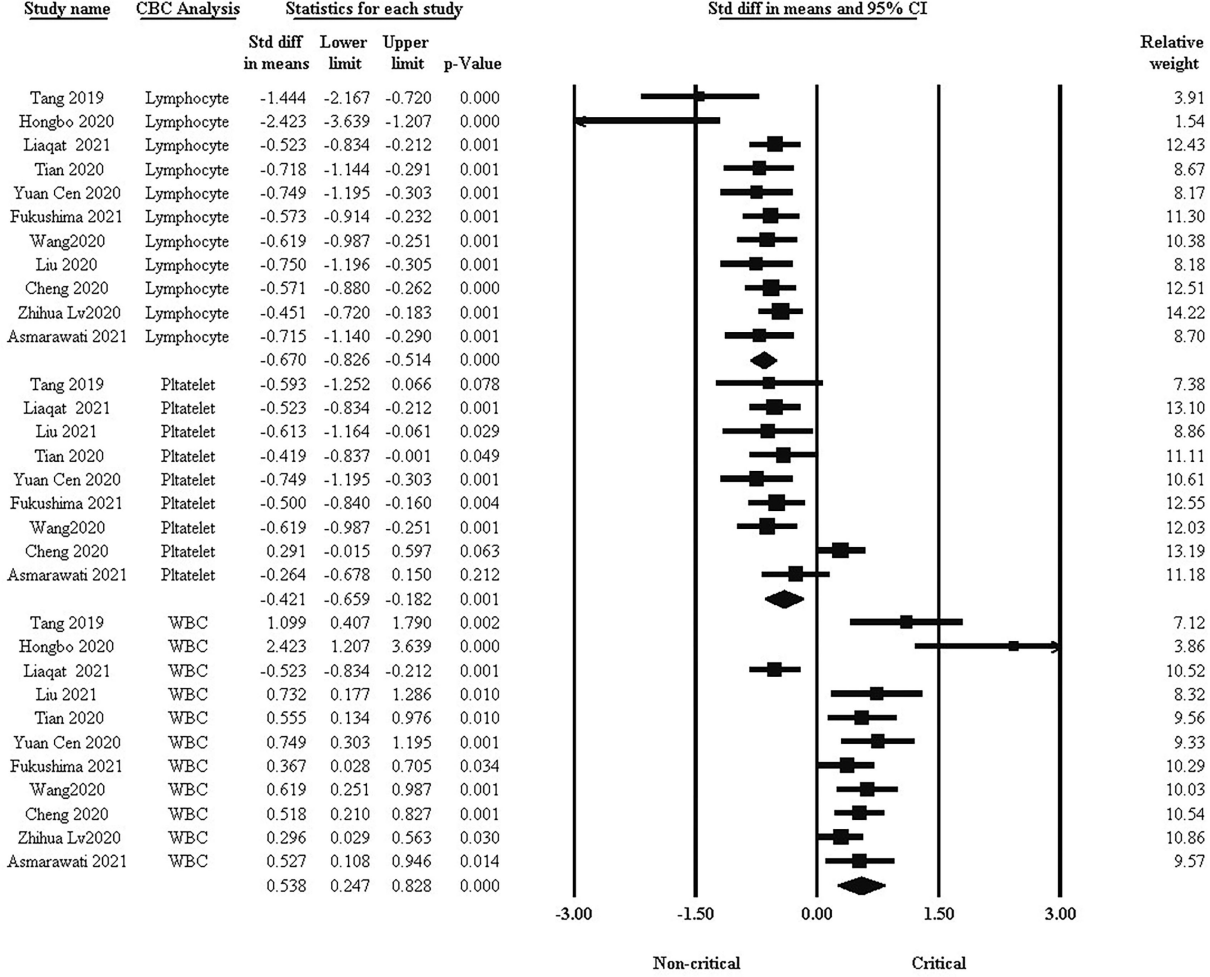
Comparison of blood cells between critical and non-critical courses.

One of the basic factors in the clinical course of COVID-19 is blood cells. We analyzed WBC, lymphocyte, and platelet differences between the two groups. As shown in **Figure 2**, the standard difference in means indicated that lymphocytes and platelets were significantly higher in non-critical patients than in critical patients, while WBCs were higher in critical patients (std: -0.670, -0.421, and 0.538, respectively, 95% CI, *P <*0.001).

### Comparison biomarkers level between critical and non-critical groups

**Figure 3:**
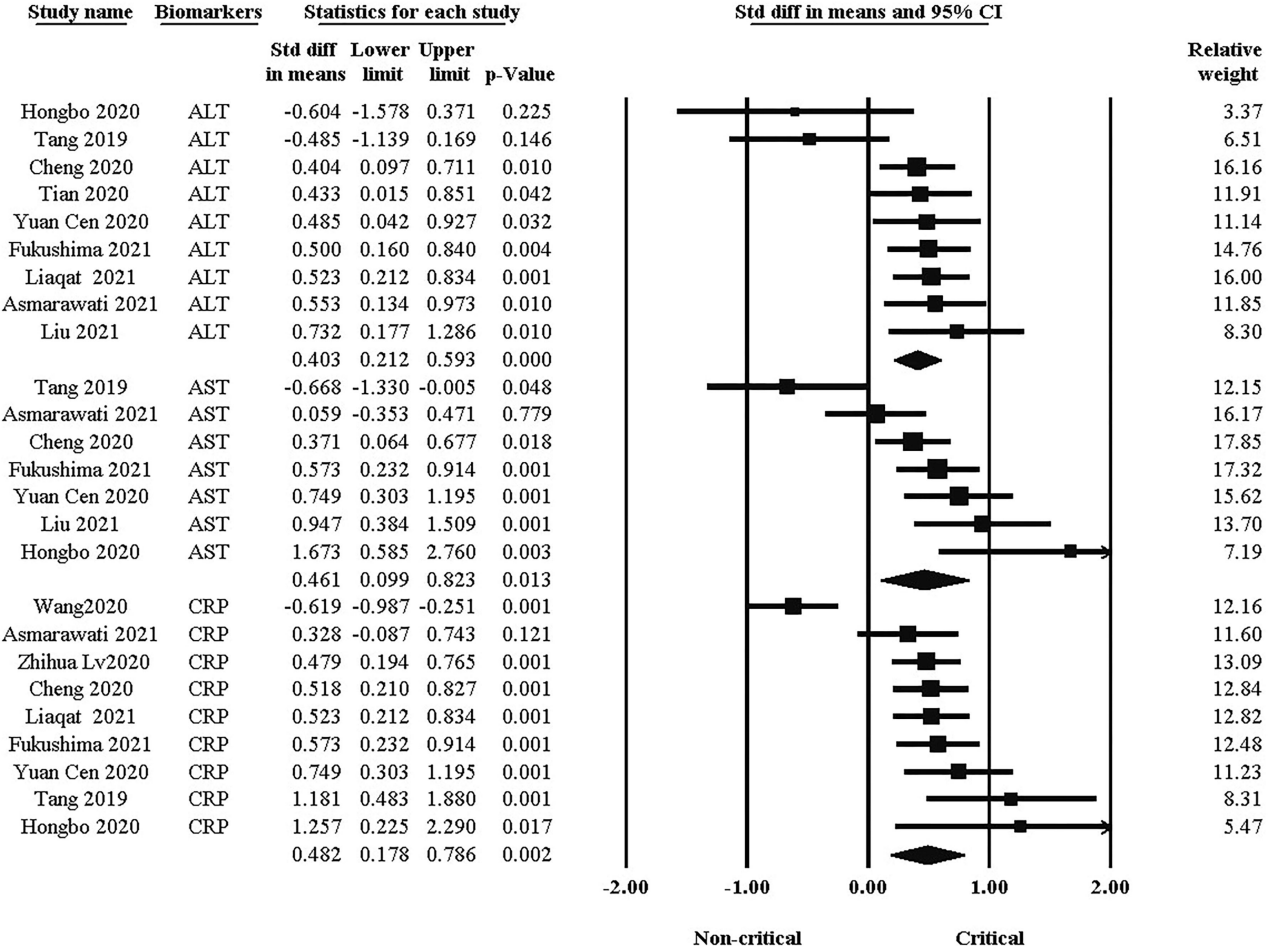
Comparison of blood markers between critical and non-critical courses.

Biomarkers and enzymes, such as CRP, ALT, and AST are other factors in the clinical course of COVID-19. As shown in **Figure 3**, there was a significant difference in blood markers between the two groups. the standard difference of the mean for ALT was 0.403 (95% CI: 0.212, 0.593. P *<*0.001), while for AST it was 0.461 (95% CI: 0.099, 0.823. P = 0.013). In addition, the standard difference of the mean for CRP was 0.482 (95% CI: 0.178, 0.786. P = 0.002). The critical group had significantly higher levels of each of these factors than the non-critical group.

### Comparison of mortality and comorbidities between critical and non-critical groups

**Figure 4:**
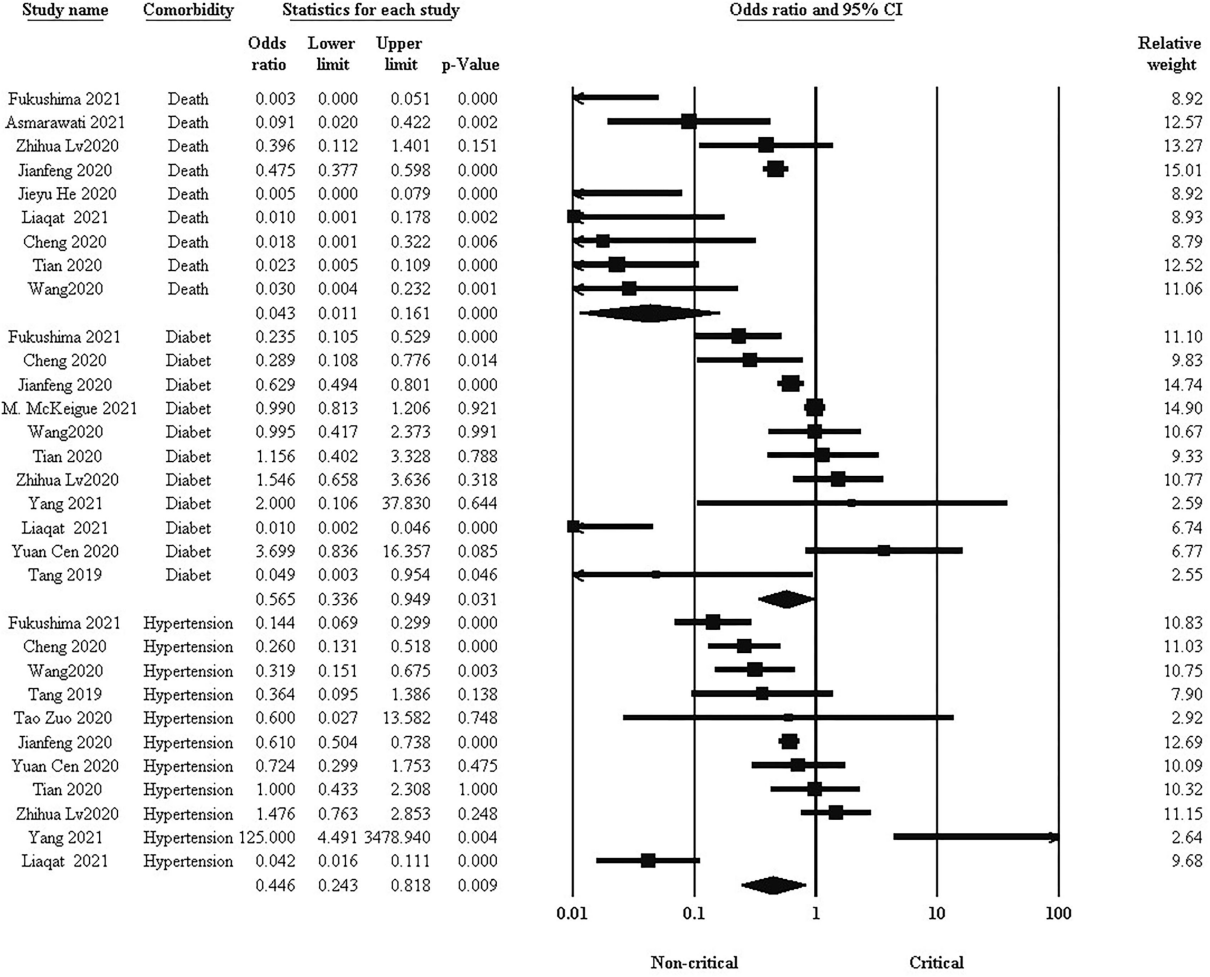
Comparison of comorbidities between critical and non-critical courses.

Comorbidities such as hypertension and diabetes are important factors related to mortality and complications of COVID-19 patients. Pooled results in **Figure 4** showed that there was a significantly higher prevalence of hypertension, diabetes, and subsequent mortality rate in the critical group ((OR: 0.446, 95% CI: 0.243, 0.818. P = 0.009), (OR: 0.565, 95% CI: 0.336, 0.949. P = 0.031), and (OR: 0.043, 95% CI: 0.011, 0.161. P < 0.001) respectively).

### Comparison of bacterial co-infection between critical and non-critical groups

**Figure 5:**
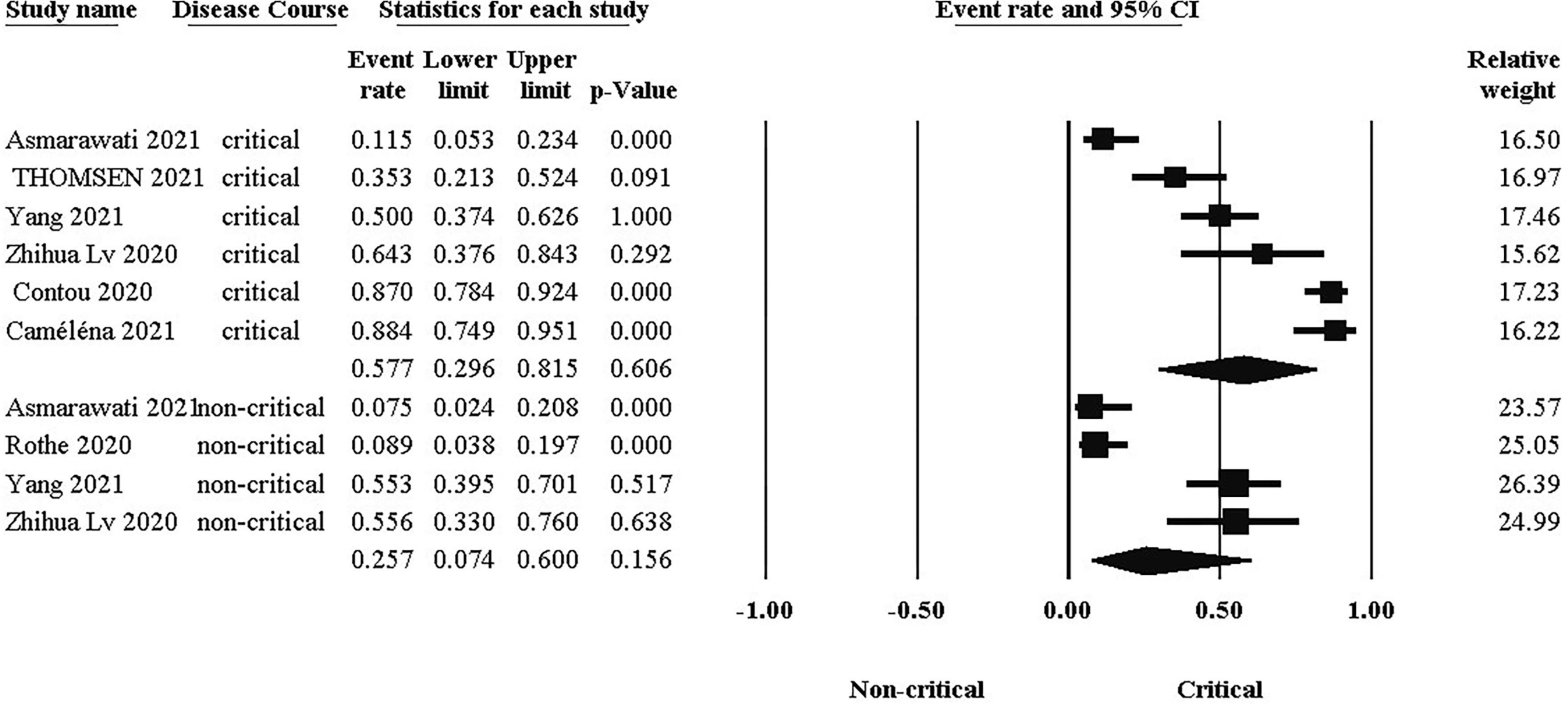
Comparison of co-infection prevalence between critical and non-critical courses.

The probability of bacterial co-infection differs significantly between groups, as illustrated in **Figure 5**. As a result, the difference in co-infection prevalence between critical (Event rate:57.7%, 95% CI: 0.296, 0.816) and non-critical (Event rate:25.7%, 95% CI: 0.074, 0.60) groups was 32%.

### Publication bias

**Figure 6:**
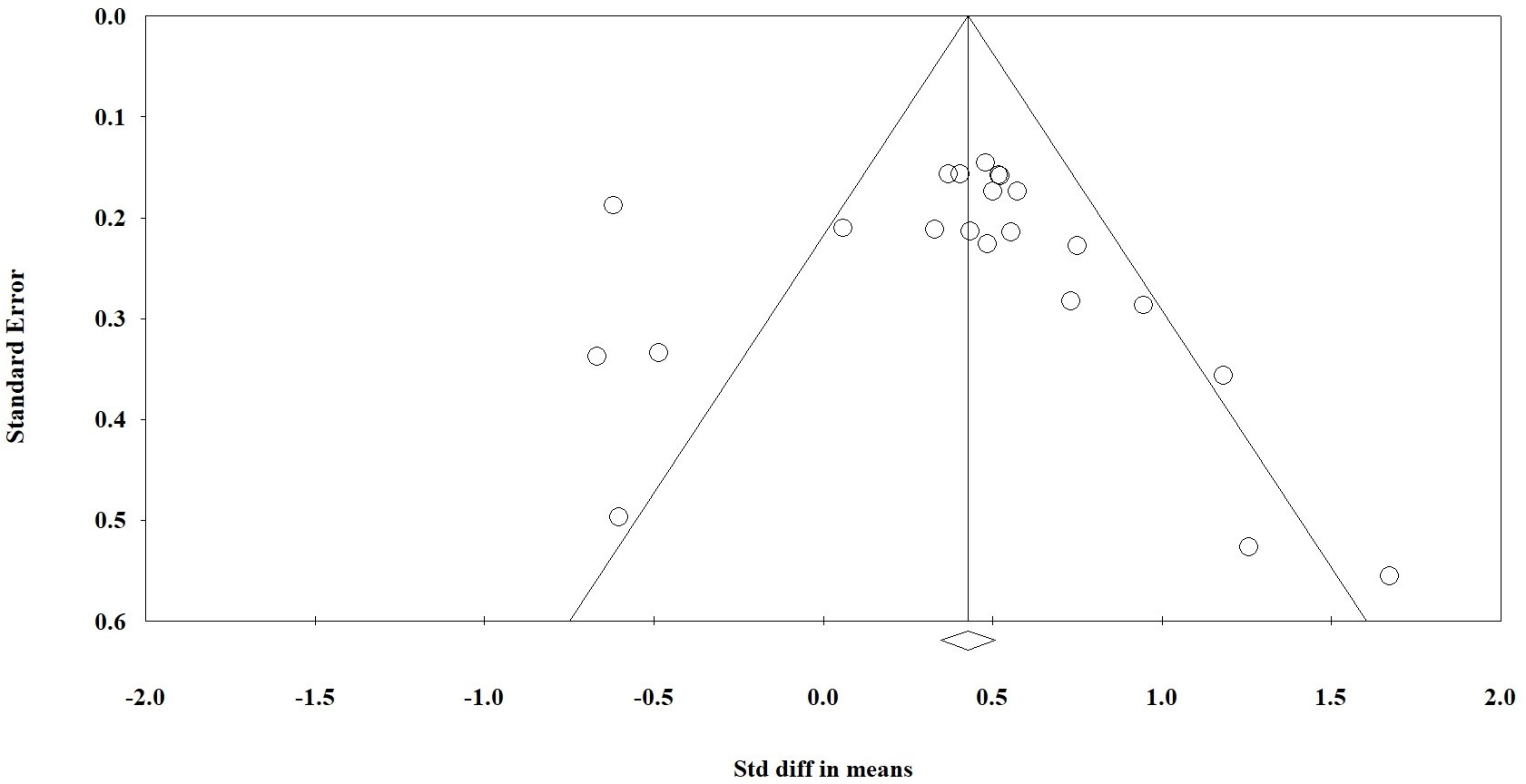
Funnel plot for comparison of blood markers between critical and non-critical courses.

A funnel plot was used for visual evaluation (**Figure 6**, 5S) and Egger’s test was used to determine bias (**Table 2**). Egger’s test indicated publication bias for three of the ten variables. According to Egger’s test, we found significant bias in WBC and lymphocyte mean and mortality rate differences between the two groups. Using **Table 2**, it appears that variables with a *P value < 0.05* are heterogeneous in terms of heterogeneity analysis. Although heterogeneous data does not necessarily indicate bias, the Egger test must be significant (*P< 0.05*). We have also attached the results of one removed study plots as **Supplementary Figures 1–3**.

**Table 2:** The Complete Results of Heterogeneity and Publication Bias Examination.

Variable	Number of report/s	Standard error	95% CI		Heterogeneity			Egger’s regression	
			Lower limit	Upper limit	X^2^	*p*-value	I^2^	*P*-value	*t*-value
Mean of WBC difference between Critical and non-Critical	11	0.148	0.247	0.828	55.46	<0.001	81.96	0.023	2.72
Mean of lymphocyte difference between Critical and non-Critical	11	0.08	-0.826	-0.514	16.48	0.087	39.335	0.001	20.69
Mean of platelet difference between Critical and non-Critical	9	0.122	-0.659	-0.182	25.4	0.001	68.54	0.24	1.28
Mean of ALT difference between Critical and non-Critical	9	0.097	0.212	0.593	13.9	0.084	42.45	0.062	2.21
Mean of AST difference between Critical and non-Critical	7	0.185	0.099	0.823	24.70	0.001>	75.71	0.72	0.36
Mean of CRP difference between Critical and non-Critical	9	0.155	.0178	0.786	41.74	<0.001	80.83	0.42	0.84
Hypertension event difference between Critical and non-Critical	11	–	0.243	0.818	69.179	<0.001	85.54	0.658	0.457
Diabetes event difference between Critical and non-Critical	11	–	0.336	0.949	64.59	<0.001	84.52	0.328	1.03
Death event difference between Critical and non-Critical	9	–	0.011	0.161	56.265	<0.001	85.78	0.0002	6.75
Prevalence of Co-infection between Critical and non-Critical cases	10	–	0.242	0.664	124.46	<0.001	92.76	0.317	1.06

## Discussion

The COVID-19 pandemic has reached a global scale, and medical systems in many countries are experiencing severe problems as a result (32, 33). The COVID-19 pandemic in 2019 has caused significant hospitalizations and deaths. According to the clinical course on COVID-19, we can classify patients with COVID-19 into critical and non-critical groups (4). The results showed that there are several differences between critical and non-critical groups. In this study, we examined the blood cell count, blood markers, and the comorbidities difference between critical and non-critical groups.

Due to the correlation between the immune system function and the clinical course of COVID-19, we compared the blood cell count between groups (34). The production of cytokines is crucial for the growth and specialization of immune cells. In COVID-19 pneumonia patients, certain inflammatory cytokines like IL-6 and IL-10 were found to be elevated in critical cases (35). However, IL-2 levels were increased in non-critical patients but decreased in critical ones. When present in low concentrations, IL-2 can prevent CD4+ T and CD8+ T-cell activation by maintaining T regulatory cell activity and survival (36). As a result, this could lead to a significant drop in CD8+ T-cells and lymphocytes in COVID-19 critical patients (37). Furthermore, critical patients had significantly lower T-cell, B-cell, and NK cell counts compared to controls (38). A gradual decrease in peripheral blood lymphocytes is a common early indicator of adult patients with non-critical and critical illnesses (39). IL-6 can stimulate T cell differentiation, and its increased levels are associated with producing acute-phase proteins like CRP and inflammatory cytokines. It is also possible that increased WBC in critical patients with low lymphocytes may be caused by an increase in PMNs, which can be indicated by an increase in CRP levels.

This study analysis indicated the higher platelet count in non-critical patients. Platelets and other related indicators play a crucial role in inflammation and prothrombotic responses during numerous viral infections (19). Apart from their traditional function in hemostasis and thrombosis, platelets also contribute significantly to the immune and inflammatory processes. Research suggests that platelets express surface receptors that enable them to bind and allow entry to various viruses. Furthermore, the rise in platelets and neutrophils could be due to anti-apoptotic cytokines and stimulation by specific pro-inflammatory cytokines (40). In addition to the immune system, enzymes and inflammation markers play an essential role in the course of COVID-19 disease (9). As a result of this study, ALT, AST, and CRP levels are significantly higher in the critical group than in the non-critical group. During acute inflammatory responses to COVID-19, there is usually a rapid and significant increase in serum CRP levels. Elevated CRP fluctuation during hospitalization has been identified as the primary cause of ICU admission with a poor prognosis (41). Analysis revealed that critical patients have higher CRP levels, indicating a more significant inflammatory response than non-critical patients (13). Although CRP is a sensitive indicator of disease activity and an independent risk factor for various diseases, studies have shown that CRP fluctuation is a better indicator of inflammation severity for guiding treatment in sepsis, systemic inflammatory response syndrome (SIRS), and community-acquired pneumonia (9, 42).

An elevated CRP level in critical patients may hint to SIRS and multi organ damage. An elevated level of ALT and AST in critically ill patients may indicate liver damage and a change in bacterial co-infection in COVID-19 disease, both of which are associated with mortality (10). Lipopolysaccharides (LPS) are always considered a major contributor to liver damage (43). In critical patients with elevated liver enzymes that are indicative of acute liver damage, LPS may be one of the contributing factors. Our analysis of critical patients reveals a high prevalence of bacterial co-infection and LPS is predominantly present in bacterial cell walls. LPS is generally released from bacterial walls during bacterial proliferation or destruction (44). Therefore, it is possible that the overused broad-spectrum antibiotics in COVID-19 patients may suddenly destroy gram-negative bacteria and induce liver damage with a large amount of LPS toxin (45). Bactericide antibiotics may cause bacteria to release LPS, so bacteriostatic are recommended instead. The bacteriostatic inhibits the proliferation of bacteria, but does not kill them, therefore the level of LPS remains low until the body can recover from COVID-19. Once COVID-19 has been eliminated, bactericide antibiotics can be used. ALT, AST, and CRP levels are associated with ICU admission risk based on the results of this study and according to the definition of critical patients.

In univariable analysis, hypertension, diabetes, cardiovascular disease, and cancer were associated with critical illnesses (24). In this study, a statistical meta-analysis revealed that comorbidities, such as hypertension and diabetes, are more prevalent in critical groups than in non-critical groups. However, some previous studies state that comorbidities are common in non-critical groups, contrary to recent studies and our meta-analysis (46). A common element of COVID-19 patients with hypertension and diabetes is the use of angiotensin-converting enzyme inhibitors (ACEI). A membrane receptor known as ACE2 is responsible for binding SARS-CoV-2 to cells and promoting its entry into the respiratory tract. The downregulation of ACE2 by SARS-Cov-2 spike protein binding reduces the protective effects of ACE2 during acute inflammation (47). ACE inhibitors may induce the ACE2 expression, the cellular receptor for SARS-Cov-2, and can aggravate the disease course (48). It has been identified that SARS-CoV-2 is able to invade cells via this previously established cell receptor which is facilitating the invasion of SARS-CoV-2 cells (49). The higher incidence of diabetes in critically ill patients can be attributed to three well-defined mechanisms (50): 1) The direct entry of viruses through various receptors in β-cells can directly cause β-cell dysfunction and apoptosis or trigger β-cell autoimmunity. Alternatively, viruses can enter pancreatic cells that express viral receptors, leading to structural and functional changes, local inflammation, and the creation of a pro-diabetic environment. This can disrupt the integrity of nearby non-infected β-cells in a paracrine manner, potentially leading to loss or dysfunction of these cells (51). 2) Targeting putative viral receptor-expressing cells in metabolic organs like the liver and adipose tissue can induce insulin resistance and result in the loss of disease tolerance mechanisms (52). 3) Induction of systemic inflammation and accumulation of prediabetic metabolites can lead to metabolic derangement and maladaptive functions (53).

Critical patients with COVID-19 pneumonia exhibit a state of immune deficiency and hypo immunity. These factors can further worsen the situation by causing severe infection and leading to fatal outcomes (54). The prevalence of bacterial co-infection in COVID-19 patients can also be another difference between critical and non-critical patients. Our meta-analysis showed bacterial co-infection is more common in critical than non-critical patients. Bronchoalveolar lavage (BAL) and sputum are usually collected in the first week of ICU admission. The majority of COVID-19 patients with bacterial co-infection previously received antibiotics. Overall, our results revealed that the frequency of bacterial co-infection is higher in critical patients following ICU admission than in non-critical patients.

Therefore, the risk of inflammation, organ damage, and previous disease is significantly higher in the critical group. According to the comparison of co-infection rates, critical patients are more likely to have co-infections than non-critical patients. Also, the critical group had a higher death rate than the non-critical group (Graphical abstract).

In conclusion, our findings suggested that critical patients have a suppressed immune system and that inflammation, organ damage, and co-infections are significantly higher. Due to these factors, critical groups have a worsened course of the disease and a high mortality rate, so these patients require rapid diagnosis and careful management. Additionally, bactericide antibiotics may cause liver failure in critical patients due to the risk of liver damage. Therefore, we suggest that this relationship be fully evaluated in future studies.

### Limitations

Incomplete and vague definitions of some articles about critical and non-critical phases.

More than three-quarters of the studies we included were from China describing patients at the start of the pandemic.

Most patients with COVID-19 patients do not require hospitalization but patients in the studies included in this review were predominantly hospitalized.

## Data availability statement

The original contributions presented in the study are included in the article/**Supplementary Material**. Further inquiries can be directed to the corresponding author.

## Author contributions

MH-C: Project administration, Resources, Supervision, Writing – review & editing. HE-S: Investigation, Methodology, Software, Validation, Writing – review & editing. AB: Conceptualization, Methodology, Writing – original draft.

## References

[1] Cevik M Bamford C Ho A . COVID-19 pandemic—a focused review for clinicians. Clin Microbiol Infection. (2020) 26:842–7. doi: 10.1016/j.cmi.2020.04.023 PMC7182753 32344166

[2] Saberi A Ghayeghran A Hatamian H Hosseini-Nejad M Bakhshayesh Eghbali B . COVID-19-associated myelitis, para/post infectious or infectious myelitis: A case report from the North of Iran. Caspian J Neurological Sci. (2020) 6:132–8. doi: 10.32598/CJNS

[3] Baghaei A Alipour N Abbaszadeh P Bakhshi A Fallah A Zeraatpishe M . Identifying and combating the Covid-19 pandemic using Artificial Intelligence. Tobacco Regul Sci (TRS). (2022), 313–30. doi: 10.18001/TRS.8.2.20

[4] Liaqat A Ali-Khan RS Asad M Rafique Z . Evaluation of myocardial injury patterns and ST changes among critical and non-critical patients with coronavirus-19 disease. Sci Rep. (2021) 11:1–8. doi: 10.1038/s41598-021-84467-4 33649391 PMC7921560

[5] Tian R Wu W Wang C Pang H Zhang Z Xu H . Clinical characteristics and survival analysis in critical and non-critical patients with COVID-19 in Wuhan, China: a single-center retrospective case control study. Sci Rep. (2020) 10:17524. doi: 10.1038/s41598-020-74465-3 33067568 PMC7567789

[6] Xu K Cai H Shen Y . Management of corona virus disease-19 (COVID-19): the Zhejiang experience. J Zhejiang Univ (Med Sci). (2022) 2:1–12. doi: 10.24205/03276716.2020.4015

[7] Shi H Wang W Yin J Ouyang Y Pang L Feng Y . The inhibition of IL-2/IL-2R gives rise to CD8+ T cell and lymphocyte decrease through JAK1-STAT5 in critical patients with COVID-19 pneumonia. Cell Death Dis. (2020) 11:1–8. doi: 10.1038/s41419-020-2636-4 32513989 PMC7276960

[8] Read KA Powell MD McDonald PW Oestreich KJ . IL-2, IL-7, and IL-15: multistage regulators of CD4+ T helper cell differentiation. Exp Hematol. (2016) 44:799–808. doi: 10.1016/j.exphem.2016.06.003 27423815

[9] Liu Z Wu D Han X Jiang W Qiu L Tang R . Different characteristics of critical COVID-19 and thinking of treatment strategies in non-elderly and elderly severe adult patients. Int Immunopharmacol. (2021) 92:107343. doi: 10.1016/j.intimp.2020.107343 33450596 PMC7833421

[10] Tang L Gu S Gong Y Li B Lu H Li Q . Clinical significance of the correlation between changes in the major intestinal bacteria species and COVID-19 severity. Engineering. (2020) 6:1178–84. doi: 10.1016/j.eng.2020.05.013 PMC7832131 33520333

[11] Choi GJ Kim HM Kang H . The potential role of dyslipidemia in COVID-19 severity: An umbrella review of systematic reviews. J Lipid Atheroscl. (2020) 9:435. doi: 10.12997/jla.2020.9.3.435 PMC7521969 33024735

[12] Tian R Wu W Wang C Pang H Zhang Z Xu H . Clinical characteristics and survival analysis in critical and non-critical patients with COVID-19 in Wuhan, China: a single-center retrospective case control study. Sci Rep. (2020) 10:1–8. doi: 10.1038/s41598-020-74465-3 33067568 PMC7567789

[13] Wang W Zhao Z Liu X Liu G Xie D Xu Z . Clinical features and potential risk factors for discerning the critical cases and predicting the outcome of patients with COVID-19. J Clin Lab Anal. (2020) 34:e23547. doi: 10.1002/jcla.23547 32860454 PMC7595899

[14] Huttner B Catho G Pano-Pardo J Pulcini C Schouten J . COVID-19: don’t neglect antimicrobial stewardship principles! Clin Microbiol Infection. (2020) 26:808. doi: 10.1016/j.cmi.2020.04.024 PMC7190532 32360446

[15] Zhang H Zhang Y Wu J Li Y Zhou X Li X . Risks and features of secondary infections in severe and critical ill COVID-19 patients. Emerging Microbes infections. (2020) 9:1958–64. doi: 10.1080/22221751.2020.1812437 PMC8284966 32815458

[16] Rahim Khorasani M Rostami S Bakhshi A Sheikhi R . Global evaluation of the antibacterial activity of Ceftolozane/Tazobactam against ESBLs-producing Escherichia coli and Klebsiella pneumoniae: a systematic review and meta-analysis. Ther Adv Infect Dis. (2023) 10:20499361231212074. doi: 10.1177/20499361231212074 38029068 PMC10656798

[17] Zuo T Liu Q Zhang F Lui GC-Y Tso EY Yeoh YK . Depicting SARS-CoV-2 faecal viral activity in association with gut microbiota composition in patients with COVID-19. Gut. (2021) 70:276–84. doi: 10.1136/gutjnl-2020-322294 PMC7385744 32690600

[18] McKeigue PM Kennedy S Weir A Bishop J McGurnaghan SJ McAllister D . Relation of severe COVID-19 to polypharmacy and prescribing of psychotropic drugs: the REACT-SCOT case-control study. BMC Med. (2021) 19:1–11. doi: 10.1186/s12916-021-01907-8 33612113 PMC7897516

[19] He J Wei Y Chen J Chen F Gao W Lu X . Dynamic trajectory of platelet-related indicators and survival of severe COVID-19 patients. Crit Care. (2020) 24:1–4. doi: 10.1186/s13054-020-03339-x 33054834 PMC7556573

[20] Cen Y Chen X Shen Y Zhang XH Lei Y Xu C . Risk factors for disease progression in patients with mild to moderate coronavirus disease 2019-a multi-centre observational study. Clin Microbiol Infect. (2020) 26:1242–7. doi: 10.1016/j.cmi.2020.05.041 PMC7280135 32526275

[21] Wu J Huang J Zhu G Wang Q Lv Q Huang Y . Elevation of blood glucose level predicts worse outcomes in hospitalized patients with COVID-19: a retrospective cohort study. BMJ Open Diabetes Res Care. (2020) 8:1–7. doi: 10.1136/bmjdrc-2020-001476 PMC7298690 32503812

[22] Fukushima K Yamada Y Fujiwara S Tanaka M Kobayashi T Yajima K . Development of a risk prediction score to identify high-risk groups for the critical coronavirus disease 2019 (COVID-19) in Japan. Jpn J Infect Dis. (2021) 74:344–51. doi: 10.7883/yoken.JJID.2020.789 33390431

[23] Liu D Lan L Luo D Zhao B Wei G He Y . Lymphocyte subsets with the lowest decline at baseline and the slow lowest rise during recovery in COVID-19 critical illness patients with diabetes mellitus. Diabetes Res Clin Pract. (2020) 167:108341. doi: 10.1016/j.diabres.2020.108341 32707212 PMC7373679

[24] Cheng S Wu D Li J Zou Y Wan Y Shen L . Risk factors for the critical illness in SARS-CoV-2 infection: a multicenter retrospective cohort study. Respir Res. (2020) 21:1–12. doi: 10.1186/s12931-020-01492-z 33087114 PMC7576549

[25] Lv Z Cheng S Le J Huang J Feng L Zhang B . Clinical characteristics and co-infections of 354 hospitalized patients with COVID-19 in Wuhan, China: a retrospective cohort study. Microbes Infect. (2020) 22:195–9. doi: 10.1016/j.micinf.2020.05.007 PMC7233257 32425649

[26] Caméléna F Moy AC Dudoignon E Poncin T Deniau B Guillemet L . Performance of a multiplex polymerase chain reaction panel for identifying bacterial pathogens causing pneumonia in critically ill patients with COVID-19. Diagn Microbiol Infect Dis. (2021) 99:115183. doi: 10.1016/j.diagmicrobio.2020.115183 33069002 PMC7441025

[27] Contou D Claudinon A Pajot O Micaëlo M Longuet Flandre P Dubert M . Bacterial and viral co-infections in patients with severe SARS-CoV-2 pneumonia admitted to a French ICU. Ann Intensive Care. (2020) 10:119. doi: 10.1186/s13613-020-00736-x 32894364 PMC7475952

[28] Rothe K Feihl S Schneider J Wallnöfer F Wurst M Lukas M . Rates of bacterial co-infections and antimicrobial use in COVID-19 patients: a retrospective cohort study in light of antibiotic stewardship. Eur J Clin Microbiol Infect Dis. (2021) 40:859–69. doi: 10.1007/s10096-020-04063-8 PMC7605734 33140176

[29] Thomsen K Pedersen HP Iversen S Wiese L Fuursted K Nielsen HV . Extensive microbiological respiratory tract specimen characterization in critically ill COVID-19 patients. Apmis. (2021) 129:431–7. doi: 10.1111/apm.13143 PMC8239678 33950572

[30] Asmarawati TP Rosyid AN Suryantoro SD Mahdi BA Windradi C Wulaningrum PA . The clinical impact of bacterial co-infection among moderate, severe and critically ill COVID-19 patients in the second referral hospital in Surabaya. F1000Res. (2021) 10:113. doi: 10.12688/f1000research 33868645 PMC8030114

[31] Yang S Hua M Liu X Du C Pu L Xiang P . Bacterial and fungal co-infections among COVID-19 patients in intensive care unit. Microbes Infect. (2021) 23:104806. doi: 10.1016/j.micinf.2021.104806 33684520 PMC7933791

[32] Zhou F Yu T Du R Fan G Liu Y Liu Z . Clinical course and risk factors for mortality of adult inpatients with COVID-19 in Wuhan, China: a retrospective cohort study. Lancet. (2020) 395:1054–62. doi: 10.1016/S0140-6736(20)30566-3 PMC7270627 32171076

[33] Bakhshi A Eslami N Norouzi N Letafatkar N Amini-Salehi E Hassanipour S . The association between various viral infections and multiple sclerosis: An umbrella review on systematic review and meta-analysis. Rev Med Virol. (2024) 34:e2494. doi: 10.1002/rmv.2494 38010852

[34] Palladino M . Complete blood count alterations in COVID-19 patients: A narrative review. Biochem Med (Zagreb). (2021) 31:030501. doi: 10.11613/issn.1846-7482 34658642 PMC8495616

[35] Azaiz MB Jemaa AB Sellami W Romdhani C Ouslati R Gharsallah H . Deciphering the balance of IL-6/IL-10 cytokines in severe to critical COVID-19 patients. Immunobiology. (2022) 227:152236. doi: 10.1016/j.imbio.2022.152236 35691133 PMC9173832

[36] Ghanbari Naeini L Abbasi L Karimi F Kokabian P Abdi Abyaneh F Naderi D . The important role of interleukin-2 in COVID-19. J Immunol Res. (2023) 2023. doi: 10.1155/2023/7097329 PMC10465260 37649897

[37] Shi H Wang W Yin J Ouyang Y Pang L Feng Y . The inhibition of IL-2/IL-2R gives rise to CD8+ T cell and lymphocyte decrease through JAK1-STAT5 in critical patients with COVID-19 pneumonia. Cell Death Dis. (2020) 11:429. doi: 10.1038/s41419-020-2636-4 32513989 PMC7276960

[38] Li X Cheng W Zhang J Li D Wang F Cui N . Early alteration of peripheral blood lymphocyte subsets as a risk factor for delirium in critically ill patients after cardiac surgery: A prospective observational study. Front Aging Neurosci. (2022) 14:950188. doi: 10.3389/fnagi.2022.950188 36118695 PMC9477480

[39] Fouladseresht H Ghamar Talepoor A Eskandari N Ghezelbash B Nejadghaderi SA Kolahi A-A . Potential immune indicators for predicting the prognosis of COVID-19 and trauma: similarities and disparities. Front Immunol. (2022) 12:785946. doi: 10.3389/fimmu.2021.785946 35126355 PMC8815083

[40] Scherlinger M Richez C Tsokos GC Boilard E Blanco P . The role of platelets in immune-mediated inflammatory diseases. Nat Rev Immunol. (2023) 23:495–510. doi: 10.1038/s41577-023-00834-4 36707719 PMC9882748

[41] Nazemi P SeyedAlinaghi S Azarnoush A Mabadi A Khaneshan AS Salehi M . Serum C-reactive protein greater than 75 mg/dL as an early available laboratory predictor of severe COVID-19: A systematic review. Immunity Inflammation Dis. (2023) 11:e1130. doi: 10.1002/iid3.1130 PMC10753867 38156391

[42] Mouliou DS . C-reactive protein: pathophysiology, diagnosis, false test results and a novel diagnostic algorithm for clinicians. Diseases. (2023) 11:132. doi: 10.3390/diseases11040132 37873776 PMC10594506

[43] Gandhi CR . Pro- and anti-fibrogenic functions of gram-negative bacterial lipopolysaccharide in the liver. Front Med (Lausanne). (2020) 7:130. doi: 10.3389/fmed.2020.00130 32373617 PMC7186417

[44] Matsuura M . Structural modifications of bacterial lipopolysaccharide that facilitate gram-negative bacteria evasion of host innate immunity. Front Immunol. (2013) 4:48636. doi: 10.3389/fimmu.2013.00109 PMC3662973 23745121

[45] Heinbockel L Weindl G Martinez-de-Tejada G Correa W Sanchez-Gomez S Bárcena-Varela S . Inhibition of lipopolysaccharide- and lipoprotein-induced inflammation by antitoxin peptide Pep19-2.5. Front Immunol. (2018) 9:1704. doi: 10.3389/fimmu.2018.01704 30093904 PMC6070603

[46] Yang X Yu Y Xu J Shu H Liu H Wu Y . Clinical course and outcomes of critically ill patients with SARS-CoV-2 pneumonia in Wuhan, China: a single-centered, retrospective, observational study. Lancet Respir Med. (2020) 8:475–81. doi: 10.1016/S2213-2600(20)30079-5 PMC7102538 32105632

[47] Gallo G Calvez V Savoia C . Hypertension and COVID-19: current evidence and perspectives. High Blood Pressure Cardiovasc Prev. (2022) 29:115–23. doi: 10.1007/s40292-022-00506-9 PMC8858218 35184271

[48] Fang L Karakiulakis G Roth M . Are patients with hypertension and diabetes mellitus at increased risk for COVID-19 infection? Lancet Respir Med. (2020) 8:e21. doi: 10.1016/S2213-2600(20)30116-8 32171062 PMC7118626

[49] Naserghandi A Saffarpour R Allameh SF . Exploring the causes of mild COVID-19 involvement in pediatric patients. New Microbes New Infections. (2020) 37:100741. doi: 10.1016/j.nmni.2020.100741 32837728 PMC7417914

[50] Lotfy MA Shama AA . Intensive insulin therapy improves the survival probability of non-diabetic COVID-19 patients presenting with acute hyperglycemia. Egyptian J Anaesthesia. (2022) 38:211–9. doi: 10.1080/11101849.2022.2060636

[51] Geravandi S Mahmoudi-Aznaveh A Azizi Z Maedler K Ardestani A . SARS-CoV-2 and pancreas: a potential pathological interaction? Trends Endocrinol Metab. (2021) 32:842–5. doi: 10.1016/j.tem.2021.07.004 PMC8302839 34373155

[52] D’Orazio MAGN . COVID-19 and obesity: overlapping of two pandemics. (2021). 6:579–85. doi: 10.1159/000518386 PMC8678214 34569546

[53] Lima-Martínez MM Boada CC Madera-Silva MD Marín W Contreras M . COVID-19 and diabetes: A bidirectional relationship. In: Clínica e Investigación En Arteriosclerosis (English Edition) (Spain: Elsevier). (2021). 151–7. 10.1016/j.arteri.2020.10.001 PMC7598432 33303218

[54] Peyneau M Granger V Wicky P-H Khelifi-Touhami D Timsit J-F Lescure F-X . Innate immune deficiencies are associated with severity and poor prognosis in patients with COVID-19. Sci Rep. (2022) 12:638. doi: 10.1038/s41598-021-04705-7 35022495 PMC8755788

