# Supplementary material for: Clinical and immunological comparison of COVID-19 disease between critical and non-critical courses: a systematic review and meta-analysis
Source: Front Immunol. 2024 Apr 16;15:1341168. doi: 10.3389/fimmu.2024.1341168 (PMC11058842; doi:10.3389/fimmu.2024.1341168)
Supplement: Supplementary file 1 [file DataSheet_1.docx]

**Supplementary Material**


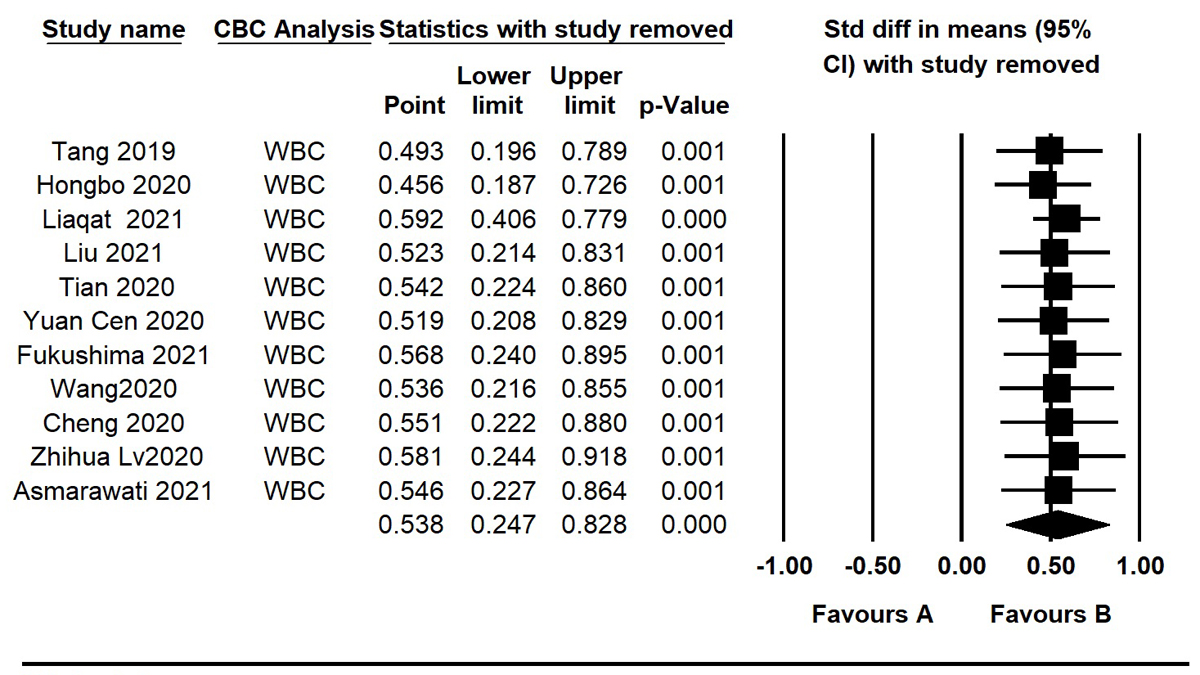


Supplementry Figure 1. One removed study plot for WBC different Meta-analysis


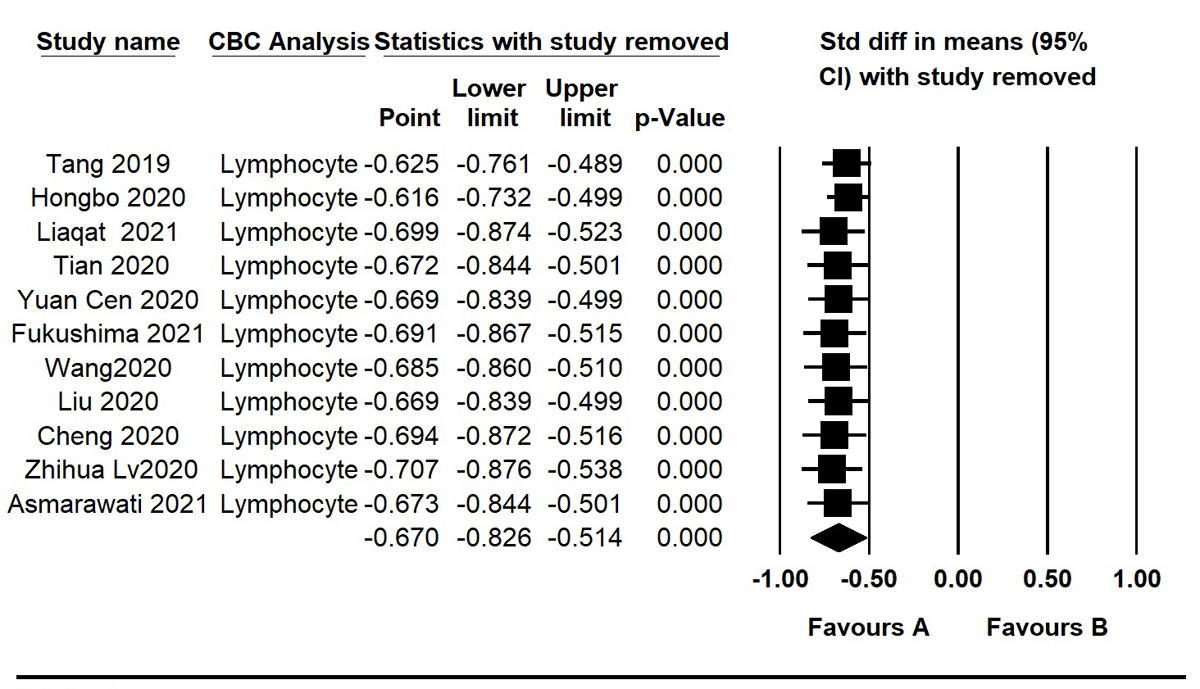


Supplementary Figure 2. One removed study plot for lymphocyte different Meta-analysis


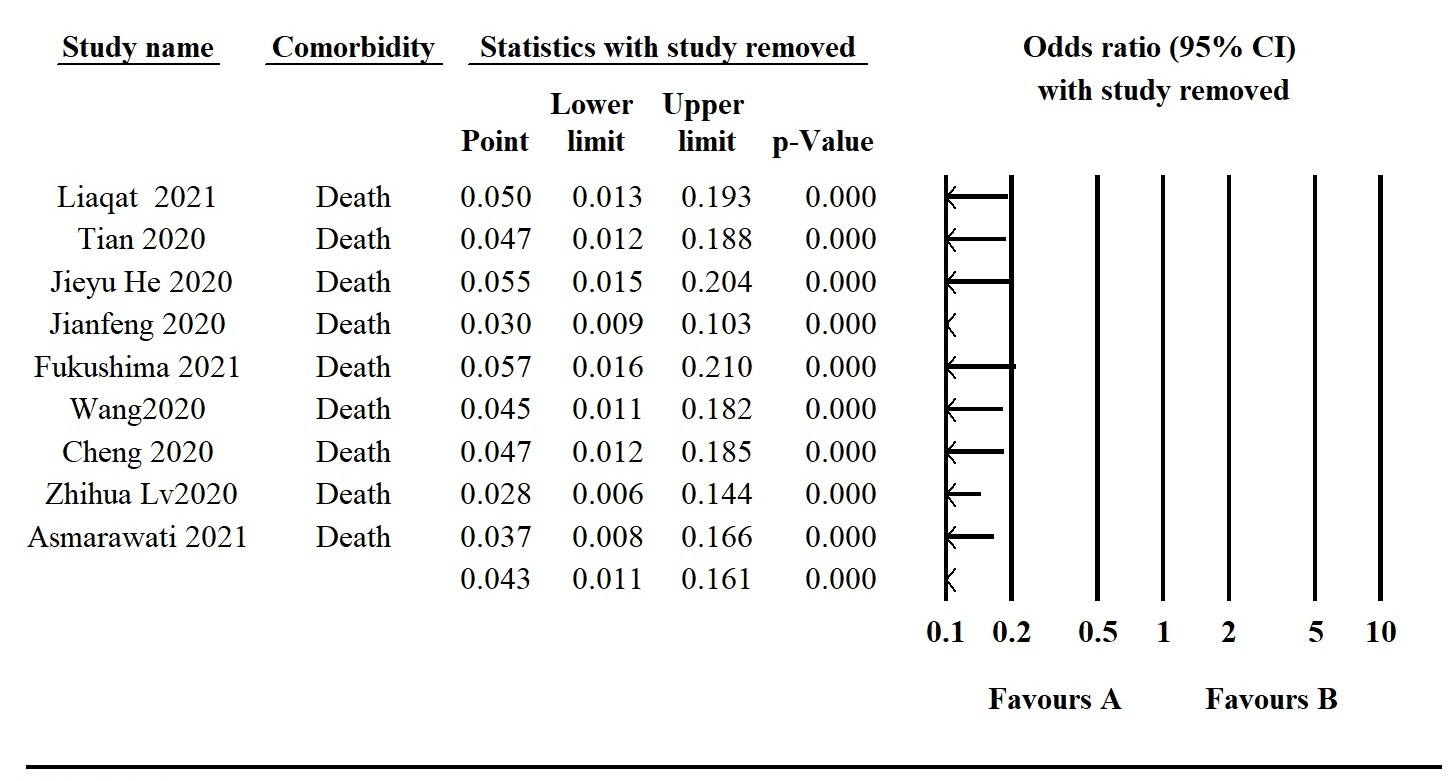


Supplementary Figure 3. One removed study plot for death different Meta-analysis


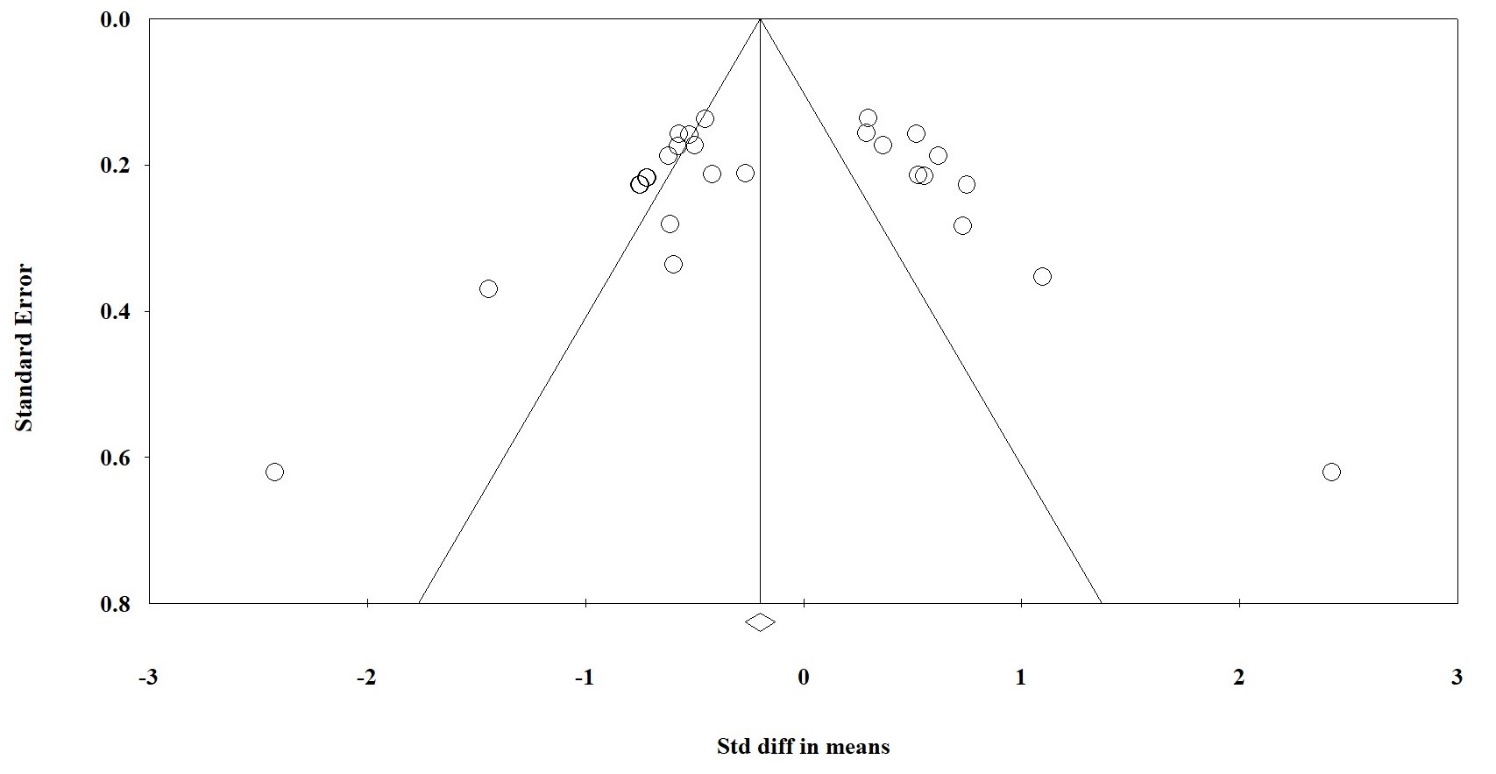


Supplementary Figure 4. Funnel plot for comparison of blood cells between critical and non-critical courses


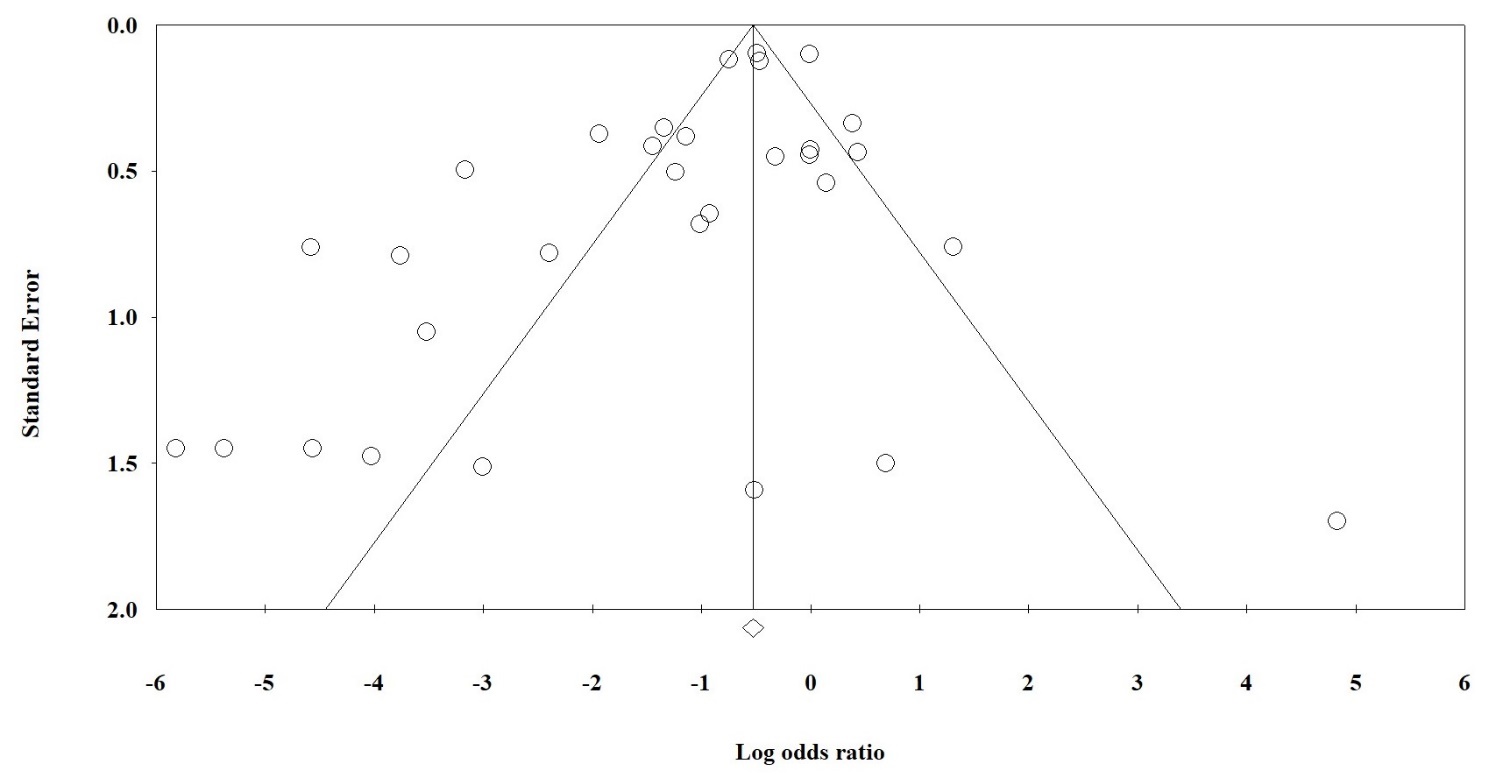


Supplementary Figure 5. Funnel plot for comparison of comorbidities between critical and non-critical courses
